# Complete Genome Sequence of *Staphylococcus succinus* 14BME20 Isolated from a Traditional Korean Fermented Soybean Food

**DOI:** 10.1128/genomeA.01731-16

**Published:** 2017-03-02

**Authors:** Do-Won Jeong, Jong-Hoon Lee

**Affiliations:** a Department of Food and Nutrition, Dongduk Women's University, Seoul, Republic of Korea; b Department of Food Science and Biotechnology, Kyonggi University, Suwon, Republic of Korea

## Abstract

The complete genome sequence of *Staphylococcus succinus* 14BME20, isolated from a Korean fermented soybean food and selected as a possible starter culture candidate, was determined. Comparative genome analysis with *S. succinus* CSM-77 from a Triassic salt mine revealed the presence of strain-specific genes for lipid degradation in strain 14BME20.

## GENOME ANNOUNCEMENT

In our previous analysis of the cultivable bacterial community during the ripening of doenjang, a traditional fermented soybean food, coagulase-negative staphylococci (CNS) species, including *Staphylococcus succinus*, were isolated as a dominant bacterial group (1). In our successive study to select potential CNS starters for Korean fermented soybean foods, *S. succinus* isolates were identified as promising candidates in terms of safety, as well as technological functionality (2). We ultimately selected *S. succinus* strain 14BME20, which did not exhibit hemolysis, biofilm formation, or phenotypic resistance to eight different antibiotics. In addition, it did not produce histamine in a laboratory setting. *S. succinus* 14BME20 grew on tryptic soy agar containing 21% (wt/vol) NaCl, exhibited acid production at 15% NaCl, and expressed protease and lipase activities. Therefore, in the current study, the complete genome sequence of *S. succinus* strain 14BME20 was determined to further confirm that it is a safe and efficient starter culture candidate for the production of fermented soybean foods.

Whole-genome sequencing was performed using a combination of the Illumina MiSeq system and the PacBio single-molecule real-time (SMRT) sequencing system by ChunLab, Inc. (Seoul, South Korea). The Illumina reads were assembled using PacBio SMRT analysis 2.3.0. Gene prediction was performed using CLgenomics (ChunLab), and sequences were annotated by comparison against the Clusters of Orthologous Groups (COG) database (3).

The complete genome of *S. succinus* 14BME20 consisted of a single circular 2,745,675-bp chromosome with a G+C content of 33.08%. The genome was predicted to contain 2,589 protein-coding sequences (CDSs), 61 tRNA genes, and 19 rRNA genes. The 2,370 genes were functionally assigned to a category based on COG assignments. Gene category analysis showed that the majority of the genes were related to amino acid transport and metabolism (247 genes; 10.4%), followed by carbohydrate metabolism (213 genes; 9.0%).

Comparative genome analysis with the previously reported genome of *S. succinus* CMS-77, isolated from a Triassic salt mine (4), revealed that 2,436 CDSs were shared between the two genomes, most of which were associated with the metabolism and transport of amino acids and carbohydrates. Although the majority of strain-specific genes were associated with hypothetical proteins, genes for lipid degradation were allocated to strain 14BME20 as functional singletons. This finding corresponds with the previously identified strain-specific lipase activity of *S. succinus* 14BME20 (2). These genes may contribute to the favorable sensory properties and volatile compound production in the fermented food products.

The high proportion of genes involved in nutrient utilization suggests the ability of *S. succinus* to persist in a large variety of habitats and degrade a range of nutrients in fermented foods. Importantly, *S. succinus* 14BME20 does not encode any of the virulence factors found in the well-known pathogen *Staphylococcus aureus*. This first complete genome sequence of *S. succinus* 14BME20 will provide further genetic insight into the safety and efficacy of this strain as a candidate starter culture for food fermentation.

### Accession number(s).

*S. succinus* 14BME20 has been deposited in the Korean Culture Center of Microorganisms under reference number KCCM 11920P. The complete genome sequence has been deposited in GenBank under accession number CP018199.
